# Molecular biomarker‐guided anti‐angiogenic targeted therapy for malignant glioma

**DOI:** 10.1111/jcmm.14417

**Published:** 2019-06-18

**Authors:** Chengrui Yan, Jiaru Wang, Yuyan Yang, Wenbin Ma, Xiaolin Chen

**Affiliations:** ^1^ Department of Neurosurgery Peking University International Hospital Beijing China; ^2^ Department of Neurosurgery, Chinese Academy of Medical Sciences Peking Union Medical College Hospital Beijing China; ^3^ Department of Neurosurgery, Beijing Tiantan Hospital Capital Medical University Beijing China

**Keywords:** anti‐angiogenic, biomarkers, glioma, molecular, therapy

## Abstract

Despite aggressive multimodality treatment, the prognosis of glioma, especially malignant glioma, remains very poor. After decades of effort, anti‐angiogenic therapy has become an important method of cancer treatment in addition to surgery, radiotherapy and chemotherapy. Although the performance of anti‐angiogenic therapy in colorectal cancer is good, its performance in malignant glioma remains unsatisfactory. Several phase III clinical trials showed no overall survival benefits. To solve this problem, the division of patients into groups based on their molecular biomarkers is an important step. This paper provides current insights into anti‐angiogenic drugs undergoing clinical trials and discusses the potential of molecular biomarkers to guide glioma diagnosis.

## 1 INTRODUCTION

Glioblastoma (GBM) is the most common and deadly brain tumour, with an annual incidence of 3.19/100 000^1^ Currently, the preferred treatment for GBM is maximal safe surgical resection followed by a combination of radiation therapy and chemotherapy with temozolomide (TMZ). Nevertheless, GBM still has a grim prognosis, with a median overall survival (mOS) ranging from 14.6 to 20.5 months.^2^, ^3^, ^4^, ^5^ The outcome is much worse in elderly patients, who have an average survival of less than 8.5 months.^6^

In 2004, the FDA commissioner announced that ‘antiangiogenic therapy can now be considered the fourth modality of cancer treatment’ in addition to surgery, radiotherapy and chemotherapy.^7^, ^8^ Angiogenesis is necessary for the growth of solid tumours and their escape from a hypoxic environment.^9^ Tumours that acquired a blood supply were observed to undergo a 19 000‐fold burst in tumour volume, which is consistent with the hypothesis of an ‘angiogenic switch’.^10^ Compared with other human neoplasms, GBM was suggested to be uniquely susceptible to angiosuppression because of its exceedingly high extent of neovascularization.^11^

VEGF and the VEGF pathway are the first targets for angiogenesis‐directed therapeutics.^12^ Vitro experiments have demonstrated that anti‐angiogenic drugs bind specifically to VEGF and prevent its interaction with its receptors. Thereby these drugs destroy pre‐existing tumour blood vessels and cutting off the supply of oxygen and other nutrients required for tumour cell growth as well as inhibiting tumour neovascularization, which in turn inhibits the growth and metastasis of tumour cells and effectively improving the transport of chemotherapy drugs.^13^ The array of clinically useful angiogenic inhibitors is now expanding beyond the VEGF pathway to include inhibitors of placental growth factor, integrin and other molecules.

Although anti‐angiogenic therapy has exhibited excellent efficacy against other human tumours, such as colorectal cancer and non‐small cell lung cancer and GBM showed high extent of neovascularization, its performance in GBM therapy is not satisfactory.^14^, ^15^ For newly diagnosed GBM, two multi‐centre, randomized phase III clinical trials (the AVAglio study and the Radiation Therapy and Oncology Group [RTOG] 0825 trial) showed benefits only in progression‐free survival (PFS), not in overall survival (OS).^16^, ^17^, ^18^ These results suggest that not all patients benefit from anti‐angiogenic therapy, and furthermore, there are no definitive biomarkers predicting the benefits of anti‐angiogenic therapy in GBM. Therefore, finding biomarkers that can identify patients who are more likely to benefit from anti‐angiogenic treatment is very important.

This article will summarize potential biomarkers that can predict the benefits of anti‐angiogenic treatment for GBM and provide diagnostic information and will describe our expectations for the near future.

## 2 BIOMARKERS FOR BEVACIZUMAB

In 1993, bevacizumab (BEV) was synthesized for the first time.^19^ As an anti‐angiogenic monoclonal antibody, BEV can slow the growth of new blood vessels in tumours by inhibiting VEGF‐A.^20^ As discussed above, two phase III trials combining BEV with radiotherapy and chemotherapy displayed no OS benefit.^17^ Molecular markers that can predict the effect of BEV on GBM therapy are urgently needed.

### 2.1 Prognostic value of molecular classification for the effect of anti‐angiogenic targeted drugs

#### 2.1.1 Isocitrate dehydrogenase 1 wild‐type proneural subtype

Better clarification of the roles of key genes has helped to classify gliomas into different molecular subtypes based on their molecular markers, providing new ideas for the clinical diagnosis and prognosis of gliomas.

Due to the heterogeneity of gliomas, patient outcome may vary across different subtypes. For example, among the four GBM subtypes in Phillips's classification,^21^ the proneural subtype was originally considered the subtype with the longest OS, but this result was later attributed to a small subset of patients with an isocitrate dehydrogenase 1 (*IDH1*) mutation.^22^ In addition, *IDH1* wild‐type proneural tumours had the worst prognosis among all GBM subtypes in the placebo arm.

Based on these facts, a retrospective study of the AVAglio trial compared the effects of BEV treatment on proneural GBM and three other subtypes of GBM (including only wild‐type *IDH1*).^16^, ^23^ The results showed beneficial effects on PFS in the proneural (9.9 vs 5.7 months; hazard ratio [HR]: 0.57; 95% CI: 0.37‐0.89; adjusted log‐rank *P* = 0.036; n = 103) and mesenchymal (10.1 vs 5.8 months; HR: 0.57; 95% CI: 0.40‐0.82; adjusted log‐rank *P* = 0.0076) subtypes. In addition, the OS was significantly longer in the *IDH1* wild‐type proneural patients (17.1 vs 12.8 months; HR: 0.43; 95% CI: 0.26‐0.73; *P* = 0.002; n = 103) (Table 1), which is consistent with the results of a previous study showing that proneural tumour cells were highly sensitive to blockage of the pathways downstream of VEGF.^24^ No significant difference in OS was observed in the mesenchymal subtype, although mesenchymal GBMs exhibit higher VEGF/angiogenic marker expression.^25^

**Table 1:** Studies assessing biomarkers in relation to the activity or efficacy of BEV

Ref.	Type	Agents	Biomarkers	Main clinical outcome
Sandmann et al^23^	Newly diagnosed GBM	BEV arm, n = 171; Placebo arm, n = 178	IDH1 wild‐type proneural GBM	mOS 17.1 mo vs 12.8 mo (*P* = 0.002)
Erdem‐Eraslan et al^27^	rGBM	BEV alone CCNU alone BEV + CCNU	Gravendeel IGS‐18	mPFS 1.4 mo, 2.9 m vs 4.2 mo (*P* = 0.0004) mOS 7.9 mo, 8.3 mo vs 11.9 mo (*P* = 0.09)
Chinot et al^30^	Newly diagnosed GBM	BEV arm n = 283; Placebo arm n = 294	MMP9	mOS 18.8 mo vs 13.6 mo (*P* = 0.0009) mPFS 11.7 mo vs 5.9 mo (*P* < 0.0001)
Tabouret et al^29^	HGG	BEV + irinotecan	MMP2	mOS 11.8 mo vs 5.9 mo (*P* = 0.009) mPFS 7.1 mo vs 4.2 mo (*P* = 0.009)
Hayes et al^32^	GBM	BEV	MicroRNA profiles	mOS 21 mo vs 15 mo (*P* = 0.026)
Bertaut et al^36^	GBM	BEV + irinotecan (before classical RT + CT) Classical RT + CT	Blood baseline neutrophil count	mOS 17.3 mo vs 8.4 mo (*P* < 0.0001)
Urup et al^38^	rGBM	BEV + irinotecan	AGT	PFS (2‐fold decrease: HR = 0.75; 95% CI: 0.59‐0.94; *P* = 0.01) OS (2‐fold decrease: HR = 0.70; 95% CI: 0.54‐0.94; *P* = 0.005),
Urup et al^38^	rGBM	BEV + irinotecan	HLA‐II	Not significant
Tobias et al^39^	rGBM	BEV therapy	PTEN	mOS 7 mo vs 5 mo (*P* = 0.0117) mPFS 5.25 mo vs 4 mo (*P* = 0.009)
Zhong et al^40^	rGBM	BEV therapy (BEV alone, BEV + TMZ or BEV + irinotecan)	BEV‐induced hypertension	mOS 11.7 mo vs 4.9 mo (*P* < 0.001) mPFS 6.7 mo vs 2.5 mo (*P* < 0.001)

#### 2.1.2 IGS‐18

In addition to *IDH1* wild‐type proneural patients, IGS‐18 patients (as defined by Gravendeel) also benefited from BEV treatment.^26^ A retrospective study of the BELOB trial using gene expression profiling and RNA‐seq found that combined BEV and lomustine (CCNU) treatment significantly improved the PFS (the median PFS was 1.4, 2.9 and 4.2 months in the CCNU, BEV and BEV/CCNU arms, respectively, *P* = 0.0004) and improved the OS (the mOS was 7.9, 8.3 and 11.9 months in the CCNU, BEV and BEV/CCNU arms, respectively, *P* = 0.09) of IGS‐18 (ie, classical GBM) patients. Subsequent research on the genes and molecular pathways associated with OS improvement revealed two possible associated genes, *FM04* and *OSBPL3*. In GBM patients treated with a combination of BEV and CCNU, a higher *FM04/OSBPL3* expression level was associated with a significantly increased mOS (6.1 vs 12.4 months, *P* < 0.0001) (Table 1).^27^

### 2.2 Prognostic value of serum biomarkers for the efficacy of anti‐angiogenic targeted drugs

#### 2.2.1 Matrix metalloproteinases

Matrix metalloproteinases (MMPs), also known as matrixins, are calcium‐dependent zinc‐containing endopeptidases, which play major roles in cell behaviours such as proliferation, migration, differentiation, angiogenesis, apoptosis and host defence.^28^

A report published in *Neuro‐Oncology* 2013 showed the predictive value of serum matrix metalloproteinase 2 (MMP2) levels by investigating the relationship between recurrent high‐grade glioma (HGG) and serum MMP2 levels (Table 1).^29^ In the initial cohort (cohort 1), patients treated with BEV and irinotecan were divided into two groups according to their response. Most patients with increased serum MMP2 levels were found to be responders (10/12). Subsequent single‐variant analysis showed that the serum MMP2 level was significantly associated with PFS and OS. In addition, matrix metalloproteinase 9 (MMP9) might be associated with PFS and OS. To verify this hypothesis, the author divided cohort 2 according to their MMP2 and MMP9 levels. The results revealed that the median PFS and OS for patients with increased serum MMP2 levels were 7.1 and 11.8 months, respectively, and were significantly higher than the corresponding values in patients with low serum MMP2 levels (4.2 and 5.9 months respectively). However, no OS/PFS benefit was observed in the MMP9 group. Similar findings were observed in the GBM patients. However, in patients treated with cytotoxic agents or immunotherapy instead of anti‐angiogenic agents, low serum MMP2 levels were associated with better OS (*P* = 0.66).

A study published in 2014 found that not only MMP2 serum levels but also the combination of serum MMP9 and MMP2 levels could predict BEV response and showed an association with OS in newly diagnosed GBM patients.^16^ Based on this research, Chinot et al performed post‐hoc evaluation of the baseline serum MMP9 and MMP2 levels in 577 of 921 tumour samples in the AVAglio trial (BEV group, n = 283; placebo group, n = 294).^30^ Patients with lower MMP9 levels (1st quartile) were found to exhibit significant OS benefits, with an improvement of 5.2 months in the mOS (HR: 0.51, 95% CI: 0.34‐0.76, *P* = 0.0009; the mOS values of the study and control groups were 18.8 and 13.6 months respectively) and PFS benefits (HR: 0.36, 95% CI: 0.24‐0.54, *P* < 0.0001). Patients with higher MMP9 levels (>3rd quartile) tended to benefit more from the placebo (OR: 1.21, 95% CI: 0.80‐1.81) (Table 1). Although the MMP9 level did not show any direct prognostic value, there was a relationship between the MMP9 level and OS (*P* = 0.03). However, no predictive or prognostic value of MMP2 levels was shown in this study.

#### 2.2.2 MicroRNA

MicroRNA expression plays an important role in the tumourigenesis, infiltration and deterioration of glioma. The role of microRNA in glioma physiology and its high stability in clinical samples indicate that microRNA may be a primary candidate as a predictive biomarker.^31^ Many researchers have sought to determine the predictive value of microRNA.

A 2016 study used TCGA data to investigate the relationship between microRNA expression and the effect of BEV treatment in GBM patients.^32^ The study identified and validated the predictive value of an 8‐microRNA profile for the therapeutic effect of BEV. The responder group was defined as having a response score >0, while the non‐responder group had a score <0.

$$\begin{array}{cc} \text{Response score} & =0.055\text{E\textbackslash{}\_miR -}124\text{a}+0.309\text{E\textbackslash{}\_miR -}202+\\ & -0.184\text{E\textbackslash{}\_miR -}204+0.170\text{E\textbackslash{}\_miR -}222+\\ & -0.194\text{E\textbackslash{}\_miR -}363+-0.025\text{E\textbackslash{}\_miR -}630+\\ & -0.322\text{E\textbackslash{}\_miR -663 + 0.161E\textbackslash{}\_miR -7} \end{array}$$

The defined cut‐off response score of 0 was used to separate a total of 37 test set samples into two groups, responders and non‐responders. The OS of the responder group was significantly longer than that of the non‐responder group (mOS 21 vs 15 months, HR = 0.34, 95% CI = 0.11‐1.01, *P* = 0.026). The researchers also calculated the response scores for all 473 GBM patients in the TCGA database (treated with various regimens excluding BEV) and found no significant difference between the responder group and the non‐responder group (Table 1). These results indicated that the predictive value of the 8‐microRNA algorithm was BEV specific. Unfortunately, the 8‐microRNA spectrum did not significantly correlate with PFS and the validation of this assay will require assessment in larger cohorts.

In addition, recent evidence has shown the predictive value of microRNA‐21 and microRNA‐10b.^33^ A meta‐analysis of previous studies showed that higher expression levels of microRNA‐21 were associated with poorer outcome in GBM patients. Unfortunately, no studies have investigated the relationship between microRNA‐21 expression and BEV treatment. Further investigation showed that the serum levels of microRNA‐21 and microRNA‐10b increased after BEV treatment, which indirectly reflected the anti‐angiogenic effect of the therapy.^34^

#### 2.2.3 Peripheral neutrophil count

Preclinical studies have shown that neutrophils may promote tumour neovascularization. Therefore, a high neutrophil count was hypothesized to be associated with a better response to anti‐VEGF therapy.^35^ To address this possibility, researchers investigated the predictive role of the peripheral blood neutrophil count before BEV treatment on the efficacy of BEV.^36^

A total of 256 GBM patients have been included in the analysis since 2006 and the best cut‐off for the baseline neutrophil count was found to be 6000/mm^3^. The results showed that increased neutrophil counts were associated with worse prognoses (13.8 months [95% CI: 11.9‐15.7] vs 18.6 months [95% CI: 15.9‐21.6], *P* = 0.0032). The effect of BEV on survival in patients with high or low neutrophil counts was further explored in another study, which revealed that patients with peripheral neutrophil counts greater than 6000/mm^3^ received significant benefit from BEV treatment (mOS 17.3 vs 8.4 months, *P* < 0.0001), whereas no significant effect of BEV on survival was observed in patients with neutrophil counts less than 6000/mm^3^ (21.6 [95% CI: 18.0‐23.3] vs 15.9 months [95% CI: 6.4‐10.3], *P* = 0.7313) (Table 1). Interestingly, the predictive value of the neutrophil count disappeared after BEV treatment: in other words, BEV was able to compensate for the deleterious effect of a high neutrophil count.

#### 2.2.4 Angiotensinogen and human leucocyte antigen class II

Angiotensinogen (AGT) and all components of the renin‐angiotensin system are expressed in GBM.^37^ In addition, increased AGT expression was associated with a higher level of vascular proliferation.

In a study published in 2016, gene expression in tumour tissue was analysed in recurrent GBM (rGBM) patients who were responsive to BEV/irinotecan combination therapy. The analysis was conducted using a platform covering 800 genes to identify predictive biomarkers for BEV response in rGBM patients. Multivariant logistic analysis and Cox regression analysis were also performed for candidate genes with possible predictive value.^38^ The results showed that 82 of 158 patients were responsive to treatment. In addition, the low gene expression of *AGT* was significantly associated with prolonged PFS (twofold decrease in *AGT*: HR = 0.75, 95% CI = 0.59‐0.94, *P* = 0.01), prolonged OS (twofold decrease in *AGT*: HR = 0.70, 95% CI = 0.54‐0.94, *P* = 0.005) and better treatment response (twofold decrease in *AGT*: OR = 2.44, 95% CI = 1.45‐4.71, *P* = 0.0009) (Table 1).

In addition to *AGT*, single‐variant analysis showed that high gene expression of human leukocyte antigen (HLA) class II was significantly associated with prolonged OS (*P* = 0.03) and better treatment response (twofold increase in HLA class II: OR = 1.22, 95% CI = 1.01‐1.47, *P* = 0.04) but was not associated with PFS (*P* = 0.16). However, this association was not detected by multivariant analysis.

### 2.3 Predictive value of *PTEN* integrity

*PTEN* deficiency is a precondition for the specific expression of VEGF‐2 in gliomas. A 2014 study investigated the possibility of using *PTEN* to predict the effect of BEV treatment.^39^ After the exclusion of *IDH1* mutant patients, 28 BEV‐treated rGBM samples were divided into *PTEN*‐positive and *PTEN*‐negative groups. The results revealed that after BEV treatment, *PTEN* positivity was significantly associated with prolonged OS (mOS 7 vs 5 months, HR = 0.46, 95% CI = 0.13‐0.67, *P* = 0.017) and PFS (median PFS 5.25 vs 4 months, HR = 0.38, 95% CI = 0.09‐0.46, *P* = 0.002) (Table 1). However, this study was limited by its small sample size and its results should be further verified by larger studies.

### 2.4 Predictive value of hypertension after BEV treatment

VEGF, via binding to VEGF receptor (VEGFR), can stimulate endothelial cells to produce NO, which leads to vessel dilatation and a decrease in arterial blood pressure. BEV can inhibit VEGF signalling and indirectly lead to an immediate increase in blood pressure. Therefore, hypertension can give indirect information about the effect of BEV and might be a prognostic factor for treatment.

An article published in *Cancer* 2014 reported the interesting finding that drug‐induced hypertension might have predictive value for the effect of BEV treatment in rGBM patients.^40^ A total of 82 rGBM patients who received BEV therapy after standard treatment were included in the study. Patients with no history of hypertension were divided into two groups: patients with post‐BEV treatment systolic pressure >140 mm Hg or diastolic pressure >90 mm Hg were placed in the hypertensive group and others in the normotensive group. The PFS and OS for the two groups showed a marked difference (PFS: hypertensive 6.7 vs normotensive 2.5 months, *P* < 0.001; OS: hypertensive 11.7 vs normotensive 4.9 months, *P* < 0.001) (Table 1). This result suggested that drug‐induced hypertension was associated with better outcome after BEV treatment, which was supported by the results from other malignancies.

## 3 BIOMARKERS FOR CILENGITIDE

Cilengitide is the first anticancer drug targeting integrin receptors to enter phase III clinical trials. Although phase II studies suggested the efficacy of cilengitide against tumours with a methylated MGMT promoter, no OS benefit was observed in phase III trials (CENTRIC: EORTC 26071‐22072) designed to evaluate the addition of cilengitide to standard therapy in patients with methylated O(6)‐methylguanine‐DNA methyltransferase (MGMT) promoter.^41^, ^42^

### 3.1 The predictive value of MGMT methylation

In 2015, Nabors et al reported a randomized, non‐blinded multi‐centre phase II clinical trial (CORE) that was closely related to the failed phase III trial. The trial was designed to evaluate the efficacy of two doses of cilengitide on GBM patients with unmethylated MGMT promoter. A total of 265 patients were randomly assigned to standard treatment (N = 89), cilengitide treatment (2000 mg, twice a week, N = 88) or intensive cilengitide treatment (2000 mg, five times a week during weeks 1‐6, thereafter twice a week, N = 88) groups. The results showed the best mOS in the cilengitide group (16.3 months), followed by the intensive cilengitide group (14.5 months), while the standard treatment group had the worst mOS (13.4 months).^43^ Accordingly, patients with unmethylated MGMT might benefit from cilengitide.

However, a multi‐centre, single‐arm, non‐blinded phase II clinical trial in 2016 led to different conclusions. This trial evaluated the effects of cilengitide combined with uninterrupted TMZ and methamphetamine on 29 newly diagnosed patients with unmethylated MGMT promoter. Compared with the historical data, combined therapy did not relieve the condition of patients but increased adverse reactions.

### 3.2 The predictive value of αvβ3, αvβ5 and αvβ8 integrins and pSmad2 levels

A retrospective study of the 2014 and 2015 trials above examined the levels of αvβ3, αvβ5 and αvβ8 integrins and pSmad2 by immunohistochemistry. The results suggested no significant association with the levels of αvβ3 and αvβ5 with prognostic information. However, in a retrospective review of the CORE study, high levels of αvβ3 in tumour cells were significantly positively correlated with improved PFS (*P* = 0.036) and OS (*P* = 0.02). More research should focus on the relationship between the expression level of αvβ3 in tumour cells and improved prognosis.^44^

## 4 BIOMARKERS FOR ENZASTAURIN

Enzastaurin, a cyclic bisindole maleimide, is an oral serine/threonine kinase inhibitor that specifically inhibits the protein kinase C and phosphatidylinositol 3‐kinase and protein kinase B (PI3K/AKT) signalling pathways, leading to cell apoptosis, the inhibition of cell proliferation and anti‐tumour‐induced angiogenesis.^45^, ^46^ In 2006, the EU and FDA approved enzastaurin for the treatment of GBM.^47^ In 2010, a phase I/II clinical trial showed that enzastaurin exhibited some anti‐tumour activity against recurrent gliomas, but it cannot be used for monotherapy.^48^

### 4.1 The predictive value of phosphorylated glycogen synthase kinase‐3β

Phosphorylated glycogen synthase kinase‐3β (pGSK3β), which may be an effective biomarker for enzastaurin, was discovered by the detection of GSK‐3 phosphorylation in peripheral blood mononuclear cells in a phase I/II clinical trial in 2010. The results indicated that the levels of pGSK3β in 20 patients decreased as the duration of treatment increased and the linear mixed model test showed that pGSK3β decreased linearly from the start to the 6th week of treatment (*P* = 0.01). However, the sample size was too small to estimate the relationship between pGSK3β and prognosis.^48^

## 5 DISCUSSION

Bevacizumab and other anti‐angiogenic drugs are effective against many advanced tumours, such as metastatic renal cell carcinoma and metastatic rectal cancer. However, the current situation regarding glioma is difficult. Although anti‐angiogenic drugs have achieved a degree of success in other tumours, their effect on glioma remains unsatisfactory. Although BEV showed some beneficial effect on PFS, other anti‐angiogenic drugs have not exhibited any convincing benefits in clinical trials. Encouragingly, however, retrospective analysis of clinical trials indicates that the OS of some patients appears to have been prolonged. These patients might have something in common.

Tumour molecular biomarkers are molecules with structural abnormalities or abnormal expression levels in tumour tissue, blood or urine. They have been used in tumour diagnosis, prognosis, treatment guidance and mechanistic research. In this paper, we have summarized related studies and found that the current research on molecular biomarkers is mainly focused on BEV, including studies on molecular typing, plasma markers and other topics. Most studies have shown significant differences between the groups divided by biomarkers. Based on these results, the application of these biomarkers to clinical treatment could have exciting potential.

However, many problems remain to be solved in using molecular biomarkers to predict the therapeutic effects of anti‐angiogenic drugs. The first problem is the heterogeneity and dynamic changes in tumours. Ideally, the therapeutic effect can be predicted by examining tumour tissue specimens or biomarkers in the circulating blood before treatment. However, not only primary tumours may be different from metastatic tumours, but the progress and treatment of a tumour may lead to changes in the tumour's biological characteristics. In addition, tumours, especially gliomas, are highly heterogeneous: different regions of the tumour may differ in their molecular characteristics. Therefore, dynamic biomarkers must be established, which leads to another problem—repeated biopsy. Fortunately, as imaging development and research continue, the use of imaging markers, such as relative cerebral blood volume, K^trans^ and others, to predict the effect of treatment is also under development. Some low‐cost biopsies can also address this problem through the analysis of markers in the circulating blood, the urine or a combination. In addition to the problems involving markers themselves, problems in the research methods cannot be ignored. The current studies are all retrospective studies of well‐designed clinical trials, lacking independent validation. In addition, more attention should be paid to molecular markers, such as peripheral blood neutrophils and plasma MMPs. Baselines must be determined to obtain unified criteria to guide follow‐up studies.

In addition to the above problems, it is also important to account for the differences among studies. The predictive weights of different markers may be different, so a formula must be established to determine the weights of different markers.

In conclusion, although many problems remain, the use of molecular biomarkers can prolong the OS of patients and reduce their financial burden. We should pay more attention to the study of biomarkers and establish a predictive formula.

## CONFLICTS OF INTEREST

None.
